# Executive Functions in Children and Adolescents with Autism Spectrum Disorder, Grade 1 and 2, vs. Neurotypical Development: A School View

**DOI:** 10.3390/ijerph19137987

**Published:** 2022-06-29

**Authors:** Ana Gentil-Gutiérrez, Mirian Santamaría-Peláez, Luis A. Mínguez-Mínguez, Josefa González-Santos, Jessica Fernández-Solana, Jerónimo J. González-Bernal

**Affiliations:** 1 Department of Health Sciences, University of Burgos, 09001 Burgos, Spain; agentil@ubu.es (A.G.-G.); mjgonzalez@ubu.es (J.G.-S.); jfsolana@ubu.es (J.F.-S.); jejavier@ubu.es (J.J.G.-B.); 2 Department of Educational Sciences, University of Burgos, 09001 Burgos, Spain

**Keywords:** autism, ASD, children, school, executive functions, BRIEF-2

## Abstract

Background: Autism spectrum disorders are neurodevelopmental disorders characterized by deficits in social and communication functioning. Previous studies suggest that people with autism spectrum disorders have deficits in executive functions, having found a relationship with cognitive flexibility, planning, working memory, inhibition or self-control, but it is especially with respect to cognitive flexibility where the greatest dysfunctions have been found. The objective of this research was to compare the executive functioning of a group of children and adolescents diagnosed with autism spectrum disorders with another with neurotypical development in an educational context. Methods: This was a cross-sectional, descriptive and multicenter confirmatory study in which 121 people who participated acted as informants, with 70 of them being education professionals who work with people with autism spectrum disorders grade 1 and 2 and 51 of them being teachers who work with people of neurotypical development; these individuals were selected through non-probabilistic sampling. Results: People diagnosed with autism spectrum disorders obtained significantly higher scores on the Behavior Rating Inventory of Executive Function-2 scale for the nine clinical scales and the four indexes that compose it compared to the group of people with neurotypical development; in addition, the average scores obtained are clinically significant, with them being elevated for the group with autism spectrum disorders. This study confirms that children and adolescents with autism spectrum disorders experience greater difficulties with respect to their executive functions than children with neurotypical development.

## 1. Introduction

The classical concept of autism has evolved substantially since its first description as a syndrome [1]. In the latest edition of the American Psychiatric Association’s Diagnostic and Statistical Manual of Mental Disorders, autism spectrum disorder (ASD) is included within neurodevelopmental disorders. It is characterized by the presence of persistent difficulties in communication and social interaction and by the presence of repetitive and restricted patterns of behavior, interests or activities [2]. Cognitive dysfunctions in people with autism spectrum disorders range from difficulties in elementary sensory processing to differences in complex social cognition [3]. Likewise, this neurodevelopmental disorder causes individuals to present social and communicative functioning deficits [4].

ASD is considered to be a specific personal continuum that debuts in childhood and is related to all of the developmental areas. The levels of support needed in all these areas are very significant to provide the highest degree of autonomy in activities of daily living (ADL) in all contexts [5].

According to the World Health Organization (WHO), approximately 1 out of 100 children is diagnosed with autism spectrum disorders [6], and the Centers for Disease Control and Prevention (CDC) reported that approximately 1 in 44 children have ASD. In addition, boys were four times more likely to be identified with ASD than girls [7]. It is an apparently growing condition possibly due to different causes such as greater awareness, better diagnostic instruments or an increase in diagnostic criteria [6,7,8]. It is more common in men than in women, with a 3:1 male to female ratio [9].

Autism is a condition that presents a heterogeneous phenotypic variability of associated genetic basis. Despite scientific advances, little is still known about genetic particularities or possible neurobiological or environmental alterations [3,10,11,12,13,14]. Although, for cognition, numerous theories have been postulated, the theory of mind, local processing and executive processing remain the most prominent ones [13,14].

The expression executive function (EF) began to be used in the mid-twentieth century to manifest different functions related to the frontal lobes. At present, there is no uniform agreement on the cognitive processes involved in EF, and various neurobiological, clinical, cognitive and behavioral theoretical models have been developed [15,16]. EF can be defined as interrelated cognitive skills aimed at providing adapted responses to novel situations in different contexts through behavioral control and regulation to achieve a goal [17,18,19,20]. Good executive functioning skills are associated with good learning and academic performance [18,20,21].

The first studies on executive functioning in autism spectrum disorders were compiled in a review by Pennigton and Ozonoff [22]. Empirical antecedents on the deficit of EF and ASD were grouped into cognitive flexibility, planning, working memory, inhibition or self-control [15,23]. Problems in executive development are closely related to difficulties in academic performance [24]. Empirical evidence points to a deficit in cognitive flexibility being the most significant dysfunction associated with EF [17,25,26,27,28,29,30,31] in natural environments such as schools.

Knowing the level of support required to adapt contexts to the needs of those with autism spectrum disorders [32] as well as the implementation of skills programs that minimize the challenge of executive functioning is essential to optimize their cognitive, behavioral and emotional functioning. Therefore, the assessment of EF must be as ecological as possible, including multiple processes and data collection from different activities and contexts [33,34], as a correct conceptualization is important to propose effective interventions [19]. 

EF research can provide insights into executive deficits that hinder the development of the skills necessary for adaptive behavior optimization [35]. Therefore, the objective of this study was to analyze the executive functional performance of children and adolescents with and without ASD in an educational social context.

## 2. Materials and Methods

### 2.1. Study Design

This research was conducted in a quantitative paradigm, with an instrumental, and non-experimental design. It was a transversal, descriptive and multicentered study. The data were obtained between September 2021 and January 2022 through a questionnaire distributed to different educational centers, both concerted and privately educational, as well as to non-profit organizations that work with people with ASD in the center-north of Spain: Burgos, Cantabria, Madrid, Palencia, Salamanca, Valladolid and Vizcaya; those who allowed to send the questionnaire to the target population.

The questionnaire collected information on the frequency of behaviors of children and adolescents with and without ASD in everyday situations in the social context of education, and correction was executed using the online service of the TEA-corrige (Trastorno del Espectro Autista-corrige, in Spanish) platform.

### 2.2. Study Participants

The study population consisted of professionals in the field of education as informants of children and adolescents between 6 and 18 years of age who are studying in regulated centers of compulsory primary and secondary education and post-compulsory secondary education. 

The sample was composed of 121 participants, of whom 70 were professionals in the educational field who work with people with autism spectrum disorders grade 1 and 2 and 51 were teachers who worked with a neurotypical or typically developing population. Those who filled in the questionnaires were ASD professionals in charge of training in social skills and school education. The evaluation of the typical group was made in a non-TEA school group from several schools, of similar ages, by professionals at the centers. For the choice of both a clinical and non-clinical sample, non-probabilistic sampling was used.

In relation to autism spectrum disorders, participants had a diagnosis of GRADE 1 and 2 ASD according to the DSM-5 diagnostic criteria [2].

The exclusion criteria were, on the one hand, the obtaining of a score in the questionnaire denominated as “high risk” in the scales of validity level of caution or alert and, on the other hand, a questionnaire with 10% or more unanswered questions. 

All participants accepted informed consent. The project was approved by the Bioethics Committee of the University of Burgos (Reference UBU 039/2020), respecting, at all times, the requirements established in the Declaration of Helsinki of 1975.

### 2.3. Instruments

EF behavior was analyzed through the Behavioral Assessment of Executive Function, second edition (BRIEF-2). This instrument consists of a questionnaire with a list of behaviors whose frequency must be estimated either by the person evaluated or by an informant, who in the case of the pediatric population in the school context will be an educator.

The BRIEF-2 is a specific questionnaire for children and adolescents that allows for a results profile to be obtained. It has been used in different clinical groups and reflects information on executive performance in the school context [36].

It is an individual questionnaire that is answered by education professionals, refers to the person evaluated and can be applied between the ages of 5 and 18 years.

The objective of this instrument is the evaluation of EF through nine clinical scales, four indices and three validity scales. The clinical scales are inhibition, self-supervision, flexibility, emotional control, initiative, working memory, planning and organization, task supervision and material organization. The four indices are (with the following three being general) the behavioral regulation index, the emotional regulation index, the cognitive regulation index and (with the following one being global) the executive regulation index. The three scales of validity are infrequency, inconsistency and negativity.

The questionnaire is composed of 63 items of frequency Likert response: never, sometimes, frequently, with it being it is completed in a time between 10 and 15 min. For the scale rating, T scores are used (M = 50; SD = 10), making it possible to determine whether the scores are potentially clinically significant (T ≥ 65). It provides a technical manual for application, correction and interpretation.

This tool presents a good internal consistency, with a Cronbach’s alpha coefficient ranging from 0.77 to 0.93 in previous research, and it has demonstrated its usefulness in the clinical diagnosis and evaluation of the prognosis of various disorders [36].

### 2.4. Statistical Analysis

A descriptive analysis was performed in order to express the sample’s sociodemographic characteristics. Subsequently, for the description of quantitative variables, the mean, standard deviation, was used. It was later found that the sample did not follow a normal Kolmogorov–Smirnov distribution (*p* > 0.05). Based on this, the Mann–Whitney ANOVA U was used to check if statistically significant differences appeared between the group of people with and without ASD in the context of EF.

Statistical analysis was performed with SPSS version 28 software (IBM-Inc, Chicago, IL, USA) and G *POWER software. For the analysis of statistical significance, a *p*-value < 0.05 was established.

## 3. Results

The sample was composed of 121 informants from the field of education, 70 (57.9%) of whom work with people with ASD grade 1 and 2 and 51 (42.1%) of whom work with neurotypical people, who provided information on the EF of children and adolescents between 6 and 18 years old through the BRIEF-2 questionnaire. Demographic data regarding sex and age are shown in Table 1.

The results for the participants with autism spectrum disorders show difficulties in the different dimensions with ranges with a clinically significant elevation (T ≥ 70), as in the case of self-supervision, flexibility, emotional control and the behavioral and emotional regulation indexes; a potentially clinical elevation (T 65–69) in inhibition, initiative, working memory, planning and organization, task supervision and the cognitive regulation index; and a slight elevation (T 60–64) in the scales and material organization. While the typical developmental participants do not reflect any apparent clinical significance dimension (T 0–59).

The Mann–Whitney U test showed significant differences for each of the nine clinical scales and the four indices that make up the BRIEF-2 scale between the groups of neurotypical people and people with ASD, as shown in Table 2. In all cases, people with ASD scored significantly higher than neurotypical people, indicating that people with ASD experience greater difficulty with respect to EF.

In the multivariate analysis carried out with the gender variable, tea women score more than tea men, in all factors, with significant differences in self-monitoring, planning, organization and in the behavioral and emotional regulation indices (Table 3).

## 4. Discussion

The present study proposes that research on EF can give rise to information about the deficits that hinder the development of skills with respect to adaptive behavior. Therefore, it was proposed that the executive functional performance of children and adolescents with and without autism spectrum disorders in the educational social context was analyzed.

This results of this research show that people with ASD experience greater difficulty with respect to EF than people with neurotypical development in the nine clinical dimensions and the four indexes that make up the BRIEF-2 scale.

When comparing the gender variable, among the participants with ASD, the results reflect greater difficulties in the female sex, with statistically significant differences in the self-monitoring and planning and organization scales as well as in the indices of behavioral and emotional regulation. In none of the scales and indices were the differences greater than 10 points, and they remained at the same level of qualitative interpretation (clinical elevation). Therefore, a lack of discrepancy between both groups could be suggested in this study. The results for the participants with ASD indicate a significant clinical elevation (T 81.5) on the flexibility scale, which is similar to other studies [17,25,26,27,31,37]. Difficulties in flexibility come with individual’s difficulties in adapting to changes from different perspectives with the objective of an adapted response to occupational demands, it can be considered a distinctive hallmark of the general autism phenotype [26,38]. It is possible to relate the clinical criteria of repetitive or restricted activities, interests and behaviors with a general predilection of people with autism for monotony [39]. This difficulty of providing emotional responses to changes adapted to the context is reflected in the emotional control index (T 79.5) and can lead to the use of maladaptive coping strategies. These behavioral responses can present themselves in the form of explosion or affective lability as a reflection of problems in the modulation of, and the provision of an emotional response adapted to, ADL and therefore can be a fundamental factor in executive functioning facilitation or distortion [39].

Regarding the behavioral regulation index (T 71.50), considered as an individual’s difficulty in adjusting behavior to the demands of an environment or context effectively, previous research, such as that by of Van Eylen et al. or Bausela-Herreras et al., refers to difficulties in inhibiting automatic responses such as executive processing dysfunction in people with ASD [29,40].

According to Pellicano et al., good inhibitory skills are important factors in early school learning [41]. In this line, skills that relate to self-knowledge and how one’s behavior can impact third parties, that is, the supervision of oneself (T 79), could also be a key factor for learning.

With respect to the cognitive regulation index (T 68.5), namely, the degree of difficulty with respect to effective management and solving problems concerning cognitive processes, previous studies also revealed difficulties in the areas of working memory (T 65) [27,39,41] and planning and organization (T 68) in people with ASD when compared to those with typical development [27,39].

On the other hand, as in the study by Blijd-Hoogewys et al. [17], the scores of children and adolescents with ASD are high, but not above the clinical limit in the material organization and initiative scales.

The results of this study may reflect that adequate cognitive regulation may be subjugated to appropriate emotional regulation [42] and appropriate behavioral regulation [36]. Early EF can make concrete contributions to social competences and thus to greater adaptation to the school environment [41]. Therefore, identifying and monitoring the strengths and weaknesses of each child’s EF could help teachers and other caregivers to expand their range of corrective intervention options to optimize school performance [43].

Considering that people with ASD present difficulties in both social cognition and executive functioning [44], a good conceptualization of EF is key to the design of effective and efficient preventive interventions that can encourage those with ASD to improve their academic performance and social interactions during their time at school [19,45].

It might be of scientific interest to replicate the current findings in adults with ASD by specifically examining whether clinically elevated scores are unique to the childhood and adolescent stages or are general regardless of the age range.

Although both the size of the effect (d > 0.80) [46] and the statistical power (1 − β > 0.80) were large, the sample size, i.e., 121 informants (57.9% ASD–42.1% typical development), was limited, so we must be cautious in making generalizations about the wider population of children and adolescents (age 6–18) with a diagnosis of Grade 1 and 2 ASD according to the DSM-5 diagnostic criteria [47].

Finally, regarding the limitations of the study, this study as carried out with children and adolescents with high-functioning ASD, which means that the results cannot be generalized to people with autism spectrum disorders at other cognitive levels. The sample consisted of more boys with ASD than girls (75.2% versus 24.8%). In addition, it is noteworthy that the sample was obtained under special circumstances due to the COVID-19 pandemic, which could have influenced the results due to the modification of daily routines in homes and schools. The use of self-report questionnaires such as BRIEF-2 may also be a limitation of this research, as results could be biased by the difference in the perception of the teachers; thus, this kind of questionnaire must be interpreted carefully and with caution, despite it being a validated questionnaire with good psychometric properties.

After determining the difficulties and needs of children with ASD in relation to their EF, more research is needed to determine what type of interventions are appropriate to alleviate these difficulties.

## 5. Conclusions

In conclusion, the results show that children and adolescents with ASD experience greater difficulty with respect to EF compared to those of neurotypical development in all the dimensions and indices included in the BRIEF-2.

As these deficits in EF are directly related to the manifestation of functional problems in certain occupations, in this case the academic field, they are clearly evidenced when they are objectively measured objectively.

These results are clinically relevant to the extent that they provide data on the specific needs of children with ASD from an educational point of view, as they provide evidence for the need for the implementation of adequate and adapted interventions that favor these people in the context of the school environment.

## Figures and Tables

**Table 1:** Demographic data, sex and age.

Mean Age	Male ASD	Female ASD	Male	Female
11.07 ± 3.57	59 (84.3%)	11 (15.7%)	32 (62.7%)	19 (37.3%)

**Table 2:** Comparison between groups in continuous variables. U de Mann–Whitney.

Variable	Median ASD	Median NT	Mann–Whitney U Test	Z	*p*-Value	1 − β	*d*
T Inhibition	67.50	54.00	982.50	−4.215	0.000	0.86	0.84
T Self-supervision	79.00	57.00	477.50	−6.87	0.000	1.00	1.81
T Flexibility	81.50	55.00	378.50	−7.38	0.000	1.00	1.91
T Emotional control	71.50	50.00	891.50	−4.69	0.000	0.91	0.89
T Initiative	66.00	53.00	838.50	−4.97	0.000	0.99	1.09
T Working memory	65.00	54.00	671.50	−5.84	0.000	0.99	1.26
T Planning and organization	68.00	53.00	615.50	−6.14	0.000	0.99	1.30
T Task supervision	68.00	52.00	774.00	−5.31	0.000	0.99	1.14
T Material organization	64.50	52.00	1091.00	−3.65	0.000	0.71	0.74
T Behavioral regulation index	71.50	55.00	681.50	−5.79	0.000	0.99	1.30
T Emotional regulation index	79.50	52.00	566.00	−6.40	0.000	0.99	1.48
T Cognitive regulation index	68.50	52.00	655.00	−5.93	0.000	0.99	1.41
T Executive regulation index	75.00	55.00	522.00	−6.63	0.000	0.099	1.62

ASD: autism spectrum disorder; NT: neurotypical.

**Table 3:** Variant analysis according to the sex of people with ASD.

Variable	Male	Female	*p*-Value
T Inhibition	67.034	74.545	0.088
T Self-supervision	74.729	83.091	0.034
T Flexibility	78.085	85.091	0.076
T Emotional control	68.559	77.182	0.083
T Initiative	63.525	68.091	0.194
T Working memory	66.254	68.364	0.522
T Planning and organization	67.525	76.091	0.009
T Task supervision	64.864	68.636	0.288
T Material organization	62.068	68.091	0.144
T Behavioral regulation index	71.068	80.182	0.021
T Emotional regulation index	76.136	85.636	0.039
T Cognitive regulation index	67.000	72.818	0.090
T Executive regulation index	72.712	81.455	0.018
